# Supplementary material for: An independent poor-prognosis subtype of breast cancer defined by a distinct tumor immune microenvironment
Source: Nat Commun. 2019 Dec 3;10:5499. doi: 10.1038/s41467-019-13329-5 (PMC6890706; doi:10.1038/s41467-019-13329-5)
Supplement: Supplementary file 2 — Supplementary Information [file 41467_2019_13329_MOESM2_ESM.pdf]

An independent poor-prognosis subtype of breast cancer defined by a distinct tumor immune microenvironment

Tekpli et al

Supplementary Figure 1

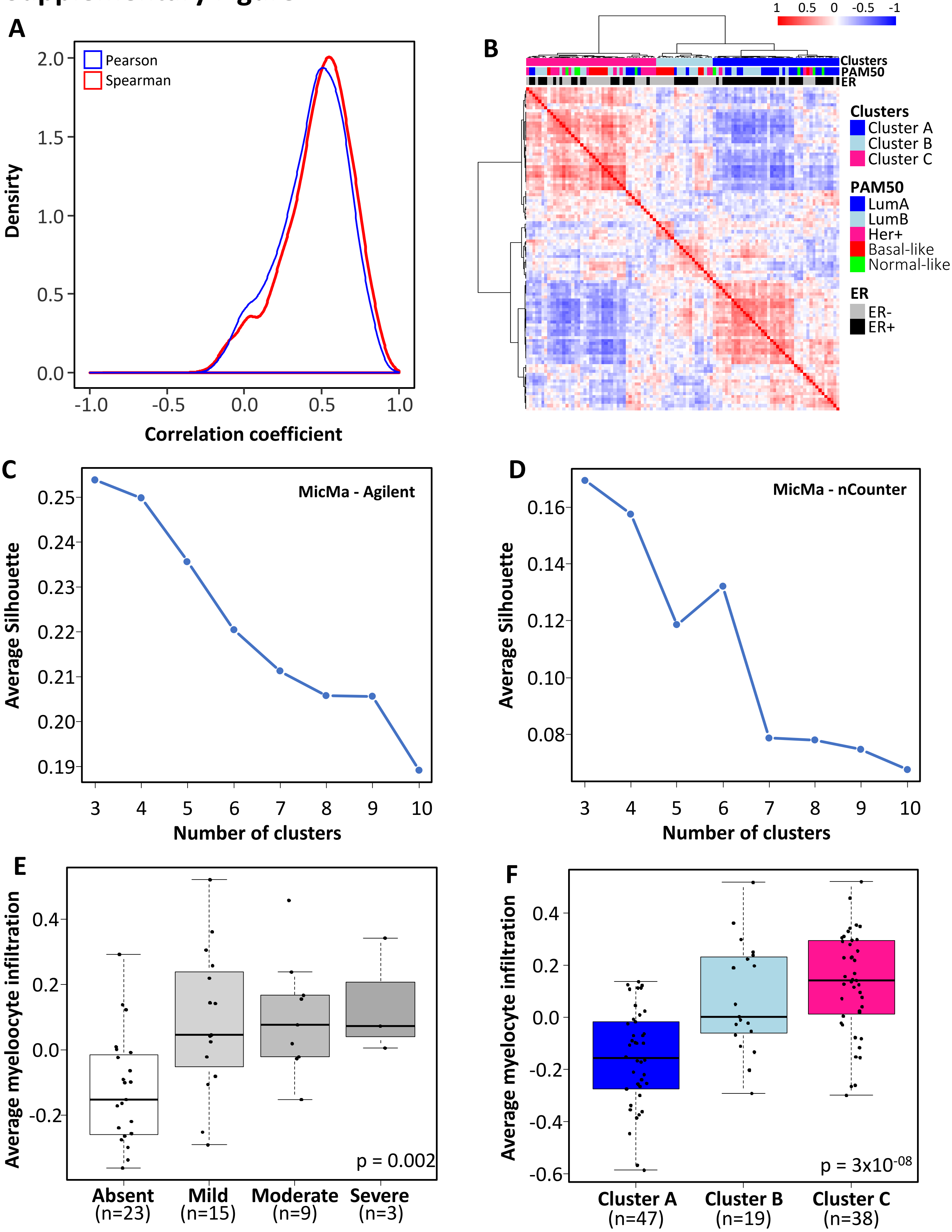

Supplmentary Figure 1: Immune clusters in the MicMa cohort

(A) Density plot showing the distribution of the Spearman and Pearson correlations of the 760 genes' expression measured by the nCounter® system or Agilent microarrays.

(B) Gene expression was measured in 104 fresh frozen MicMa samples. Unsupervised clustering using correlation distance and ward.D linkage of the correlation matrix assess the connection between patients according to the expression of 509 out of the 760 genes on the PanCancer Immune Profiling array. Annotations on the top of the heatmap indicate: PAM50 subtype, ER status and 3 clusters identified.

(C-D) silhouette plot analysis was used to determine the optimal number of clusters in the MicMa-agilent (C) and the MicMa-nCounter (D) data. The mean silhouette plot on the y-axis is plotted according to cluster combinations from 3 to 10. In both cases the mean silhouette value was higher for 3 clusters.

(E) Tumor tissue samples (MicMa, n=50) were scored for the presence of infiltrating immune cells (absent, mild, moderate, or severe) by an experienced pathologist. Boxplots represent the average myeloïd score according to pathologists' classification. Kruskal–Wallis test p-values is denoted.

(F) Levels of myeloïd infiltration were calculated in the MicMa cohort from a set of genes' markers of myelocyte as defined by the algorithm Nanodissect (*29*). Average myeloïd infiltration according to immune clusters is represented in boxplots with the Kruskal–Wallis test p-value.

Supplementary Figure 2

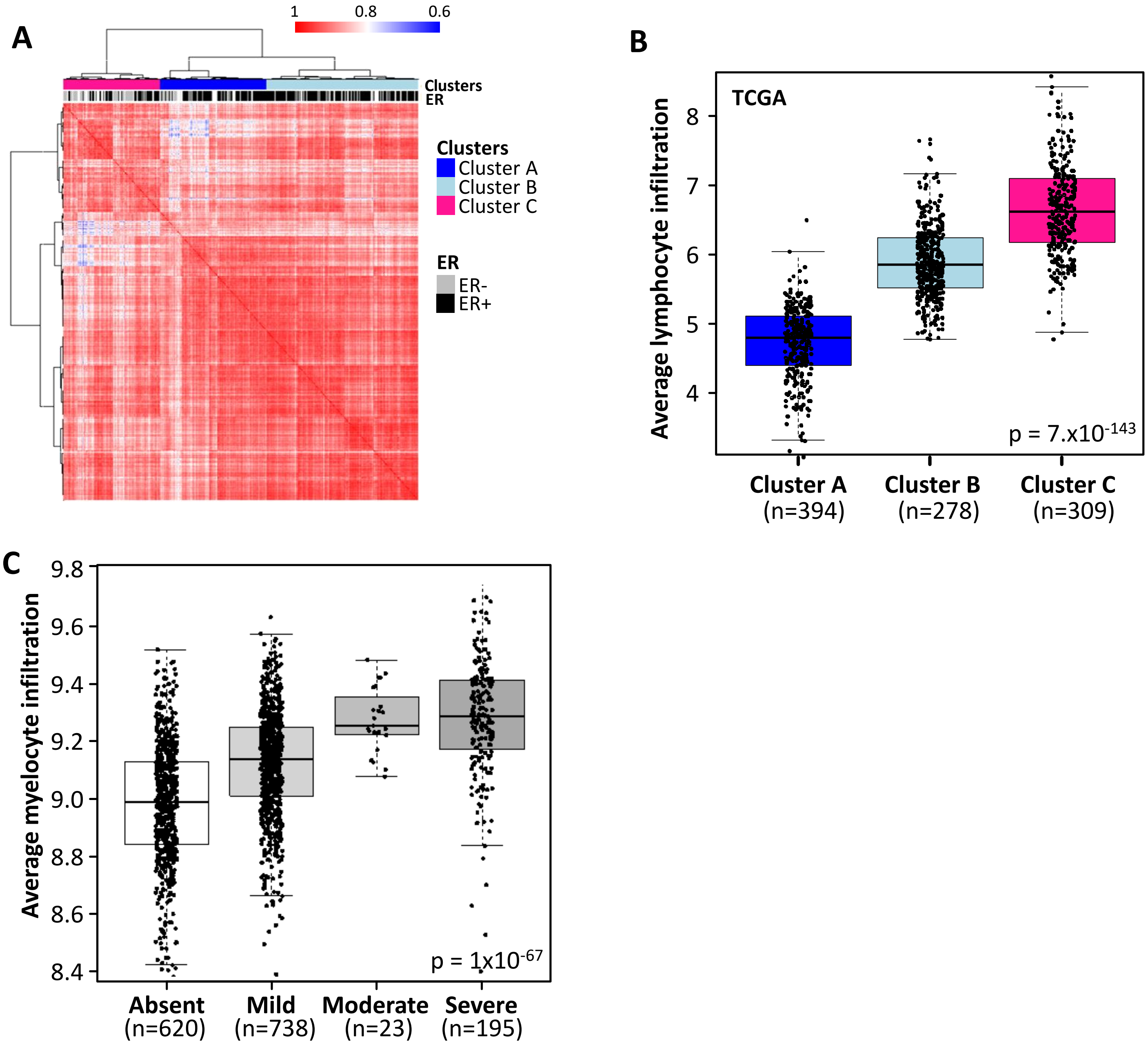

Supplementary Figure 2: Immune clusters in the TCGA cohort

(A) 509 genes' expression retrieved from BRCA-TCGA dataset were used in unsupervised clustering using correlation distance and ward.D linkage of the correlation matrix. Annotations of the patients on the top of the heatmap indicate ER status as well as the 3 clusters of patients identified by cutree.

(B) Levels of lymphocyte infiltration were calculated in the TCGA cohort using genes markers of lymphocyte infiltration defined by Nanodissect. Average lymphocyte infiltration according to immune clusters is represented in boxplots with the Kruskal–Wallis test p-value.

(C) Infiltrating immune cells (absent, mild, moderate, or severe) were retrieved for the METABRIC. Boxplots represent the average myelocyte score according to pathologists' classification. Kruskal–Wallis test p-values is denoted

Suppplementary Figure 3

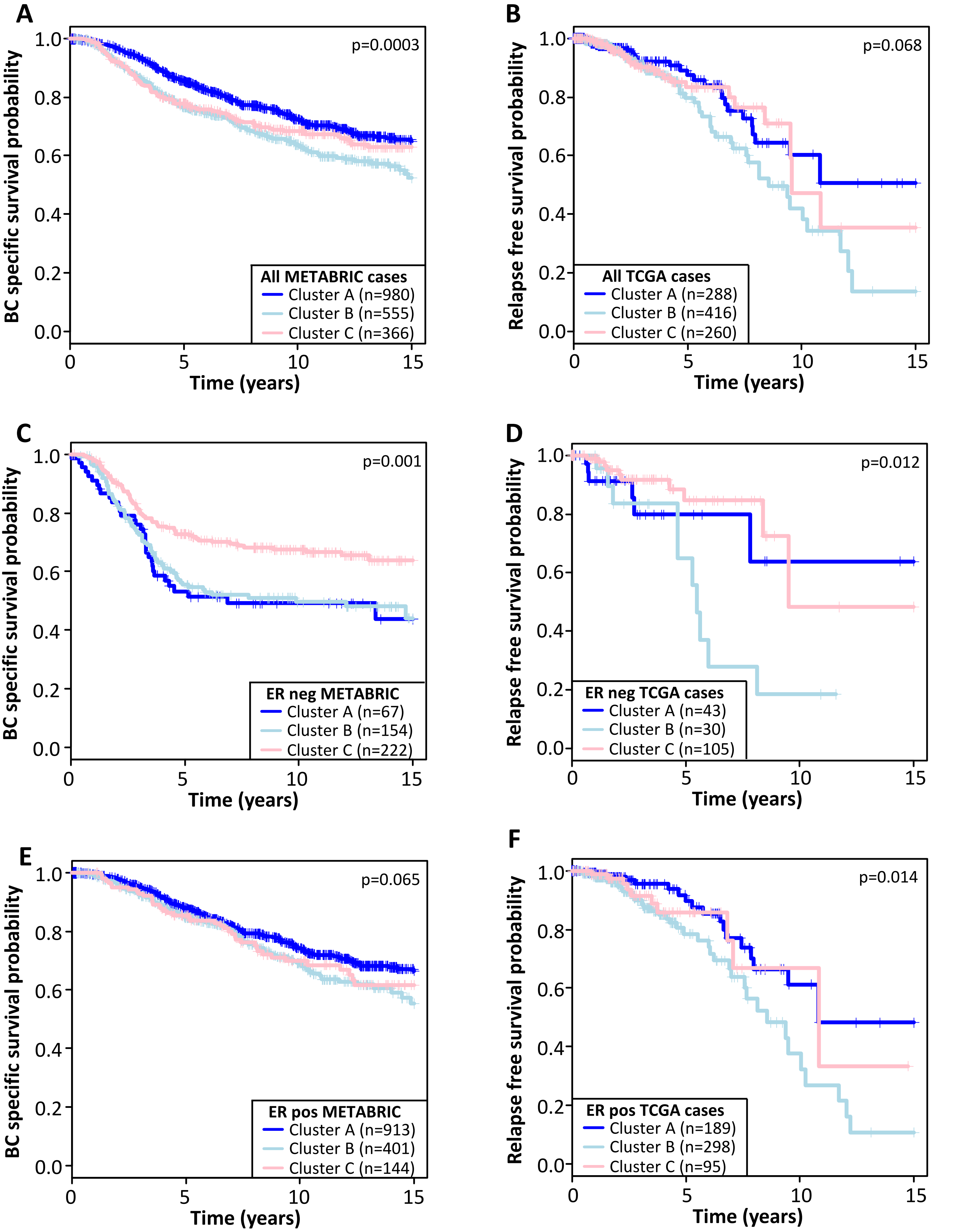

Supplementary Figure 3: Immune clusters are associated with prognosis
Kaplan-Meier survival curves for Cluster A (blue), Cluster B (light blue) and Cluster C (pink). In all METABRIC **(A)** and TCGA **(B)** samples; in ER negative (**C & D**) and ER positive (**E and F**). The p-values are from log-rank tests. Kaplan-Meier display breast cancer specific survival for the METABRIC and relapse free survival for the TCGA.

Supplementary Figure 4

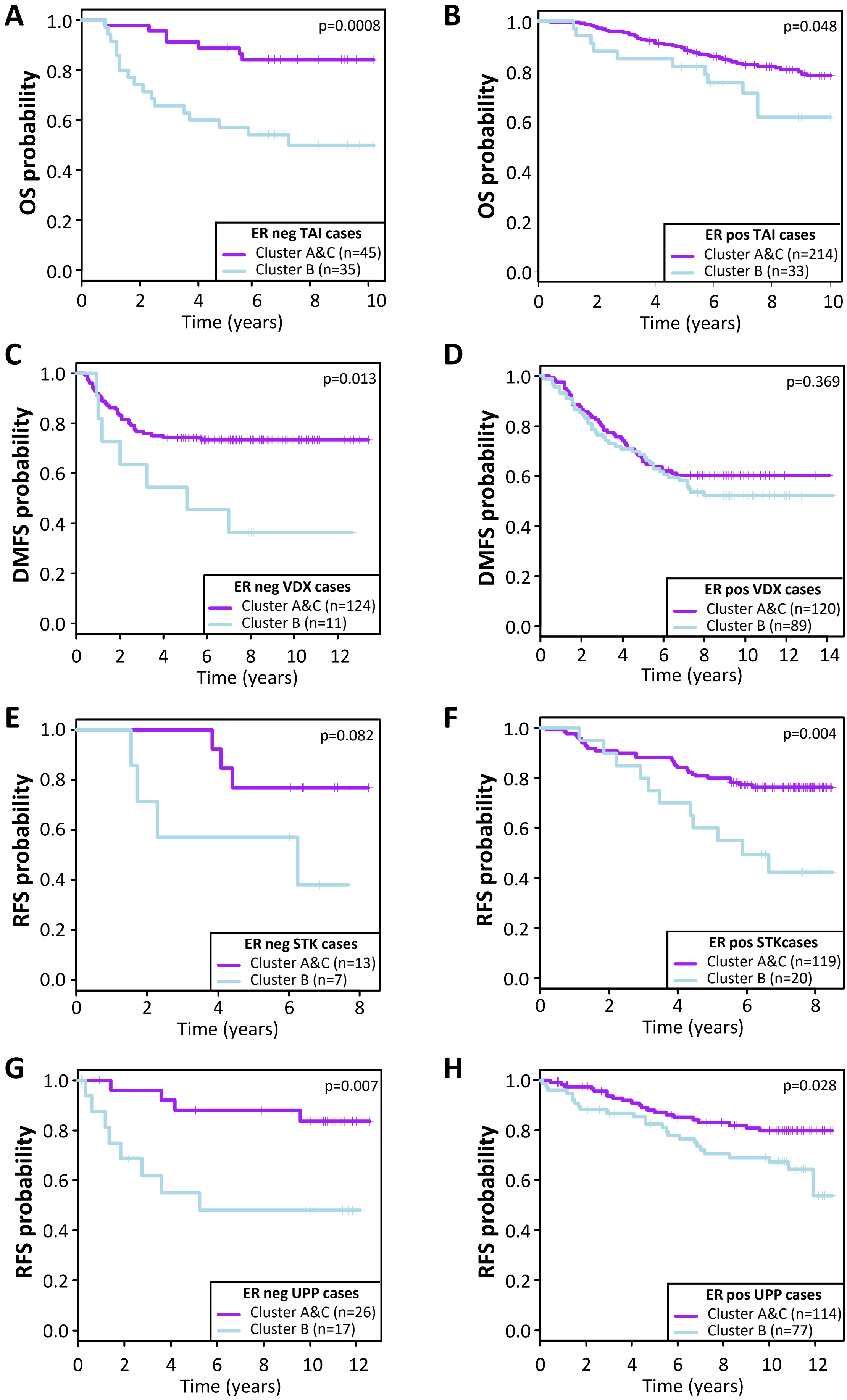

Supplementary Figure 4: Validation of the association between immune clusters and prognosis
Kaplan-Meier survival curves for Cluster B (light blue) and Cluster A & C (purple). In TAI, VDX, STK and UPP cohorts. ER negative samples (left panels, **A, C, E, G**); ER positive samples (right panels, **B, D, F, H**). p-values are from log-rank tests.

Supplementary Figure 5

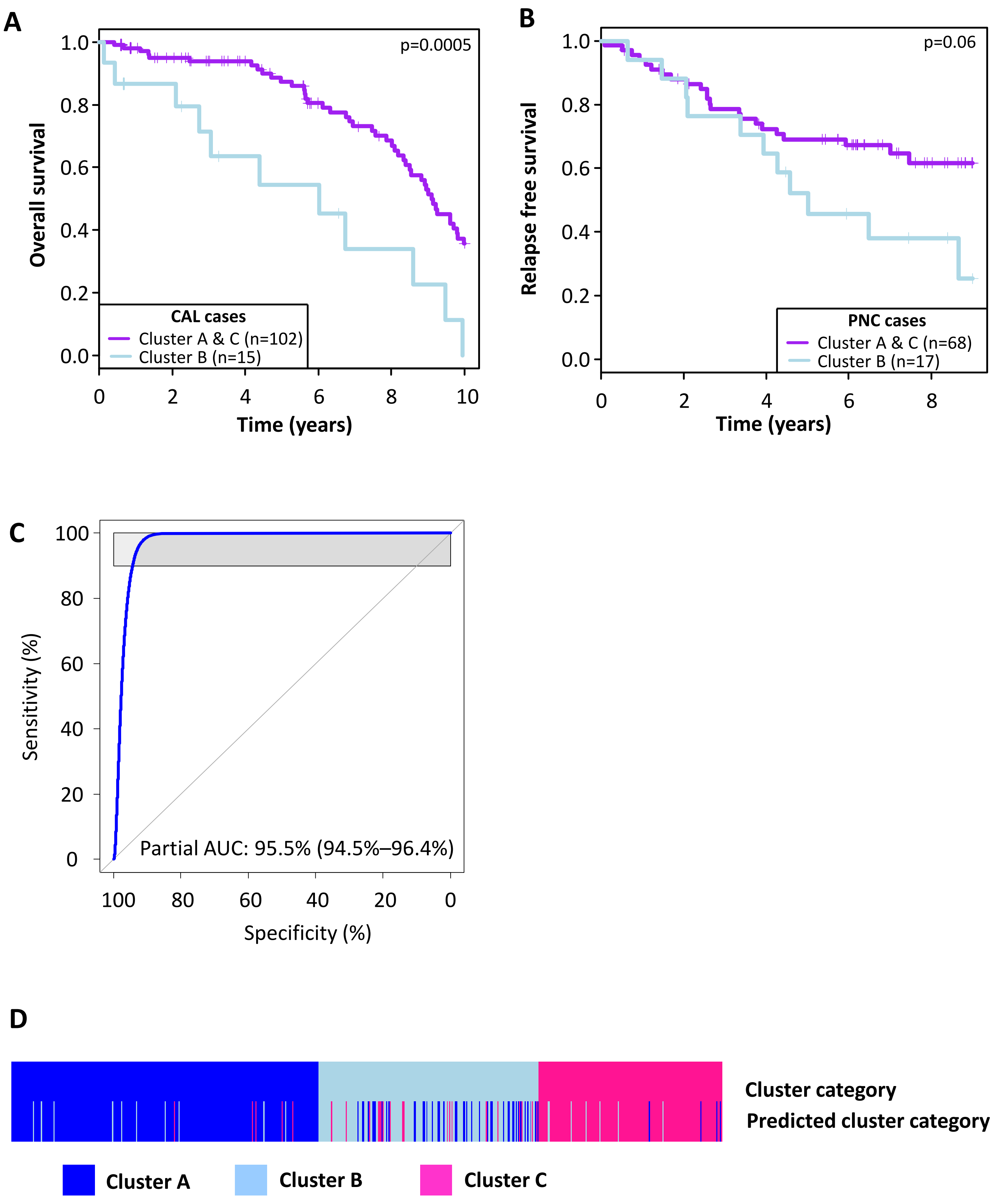

Supplementary Figure 5: Prediction of Cluster A and C using binomial logistic regression

(A & B) The prediction of the clusters (lasso) was tested on five cohorts which were not included in the training phase, three are presented in the main Figures, the two other cohorts CAL (n=118) and PNC (n=92) are presented here. The association between predicted clusters and survival was tested using Kaplan-Meier survival curves for predicted Cluster B (light blue) and predicted Cluster A & C (purple). The p-values are from log-rank tests. Kaplan-Meier display relapse free survival for STAM, distant metastasis free survival for MAINZ and overall survival for UPSA.

(C) To distinguish Between Cluster A and C a second round of binomial logistic regression penalized by the lasso method was performed. The efficiency of the model was assessed using ROC curves. The model showed extremely good performances with an area under the curve (AUC) of 95.8% (with 95% of CI between 95.5%–96.4%).

(D) Heatmap representing distribution of 4546 samples (10 cohorts included in the training phase) across the immune clusters which were determined either using the clustering or the lass method.

Supplementary Figure 6

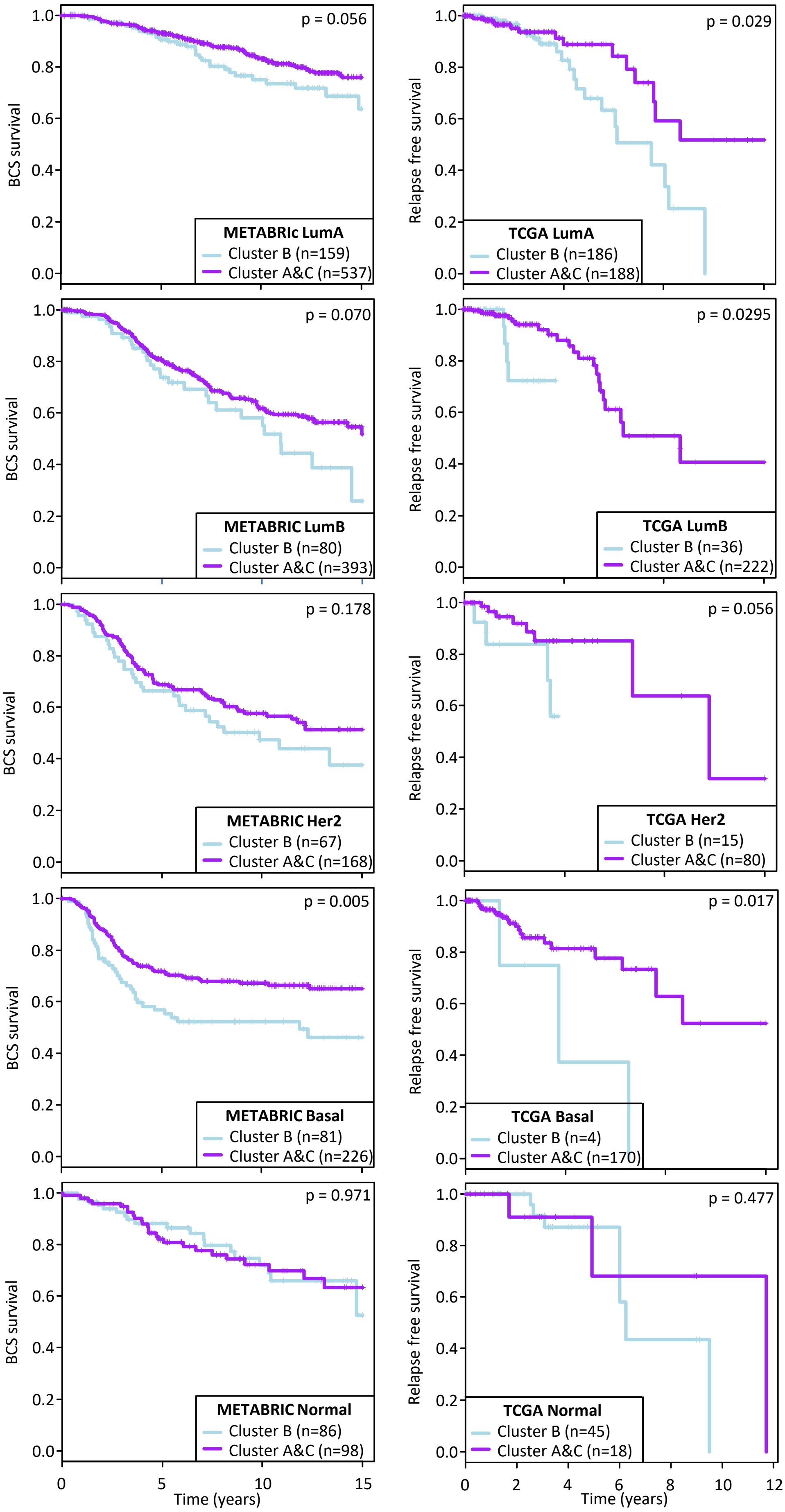

Supplementary Figure 6: Importance of the immune clusters in each PAM50 subtype
Kaplan-Meier survival curves for Cluster B (light blue) and Cluster A & C (purple). In METABRIC (left panel) and TCGA (right panel) cohorts. Luminal A, Luminal B, Her2-enriched, Basal-like and Normal-like samples were assessed independently for survival in perspective of the immune clusters. p-values are from log-rank tests. Kaplan-Meier display breast cancer specific survival for the METABRIC and relapse free survival for the TCGA.

Supplementary Figure 7

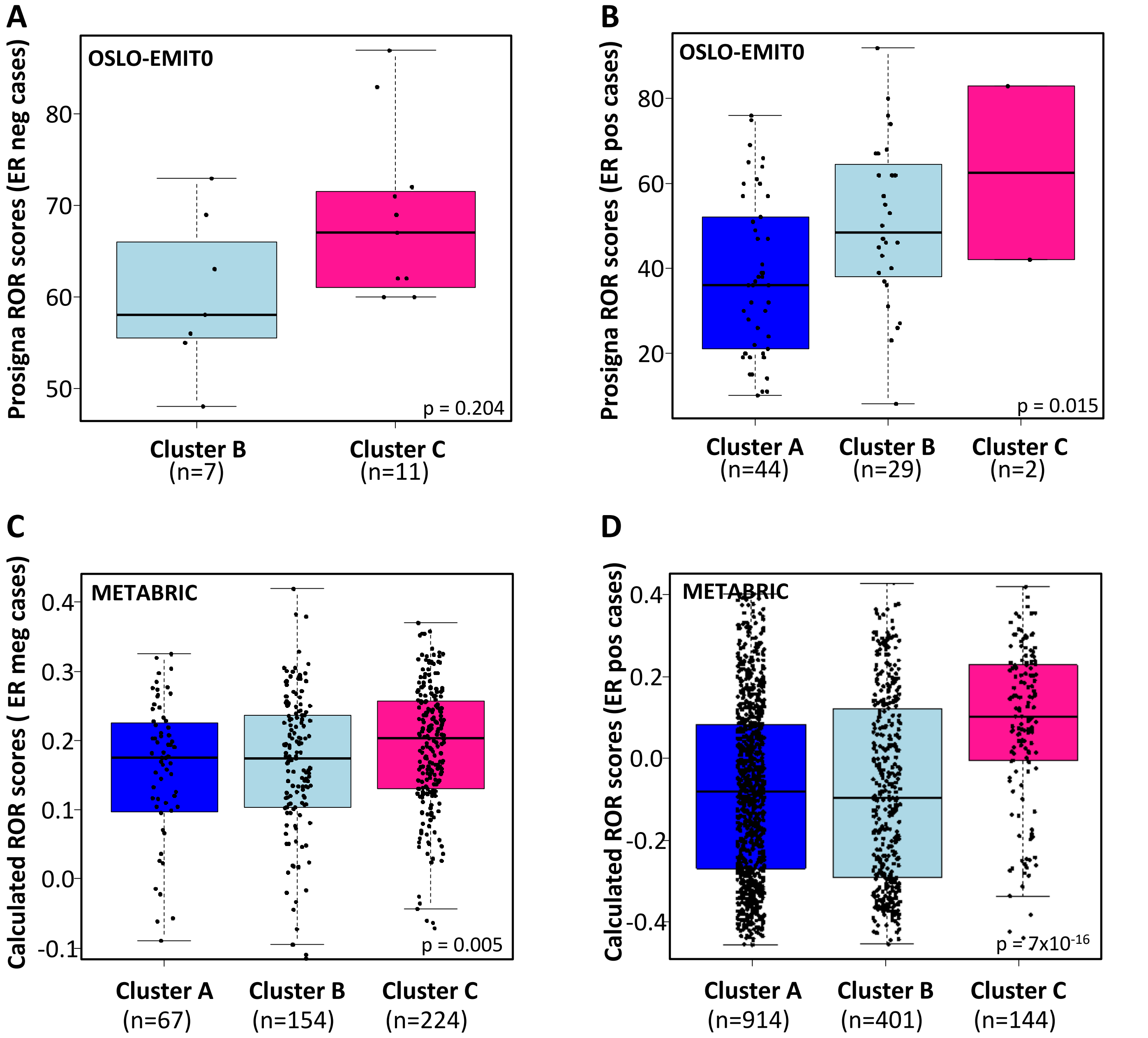

Supplementary Figure 7: ROR scores according to immune clusters and ER status

(A & B) Prosigna Breast Cancer ROR scores for the OSLO-EMIT0 cohort were obtained from the NanoString nCounter Dx Analysis System using FFPE breast tumor tissue. **(C & D)** ROR scores were calculated following the Parker's method for the METABRIC. Distribution of the ROR scores across the immune clusters in ER negative (left panels) and ER positive (right panel) are shown in boxplots. Kruskal–Wallis test p-values is shown. Note they were no ER neg samples in Cluster A for the EMIT0 explaining that for boxplot (A) only Cluster B and C are seen.

Supplementary Figure 8

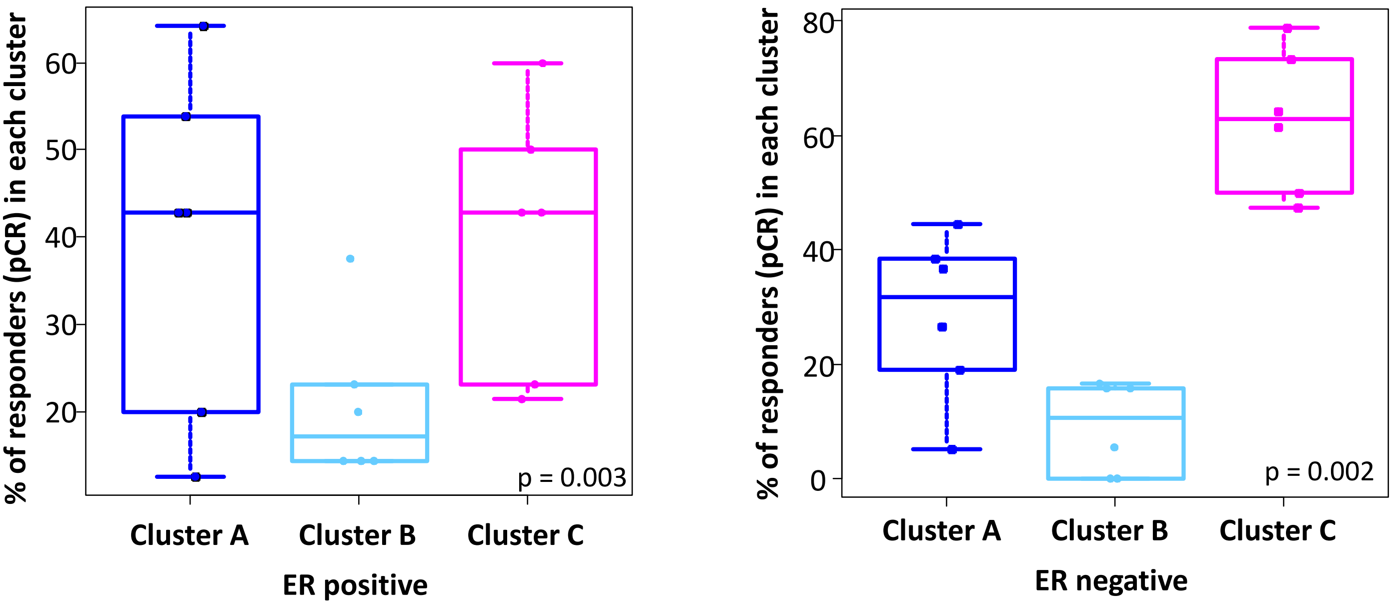

Supplementary Figure 8: Immune clusters and response to neoadjuvant chemotherapy by ER status
From 9 breast cancer cohorts, in which the pathological complete response (pCR) was assessed after administration of neoadjuvant chemotherapy, we calculated the percentage of responders in each cluster in ER positive and ER negative cases. Boxplots show the distribution of the percentage of responders in each cluster. Kruskal–Wallis test p-value is denoted.

Supplementary Figure 9

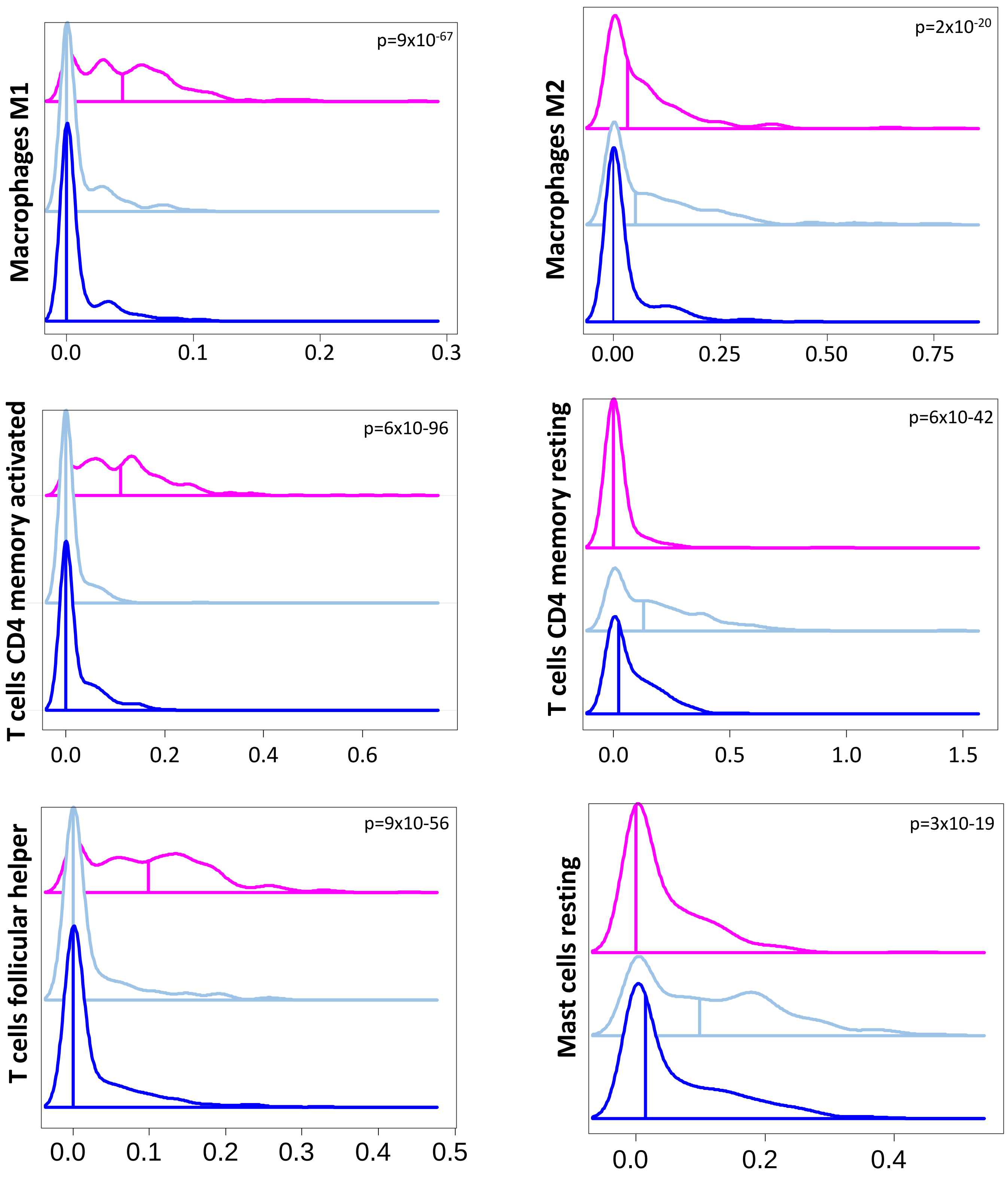

Supplementary Figure 9: Distribution of CIBERSORT scores across the clusters

Density plots represents the distribution of the absolute CIBERSORT scores for selected cell types across the clusters for the TCGA cohort the vertical lines crossing the distribution identify the median value for the scores. Kruskal–Wallis test p-value are denoted.

Supplementary Figure 10

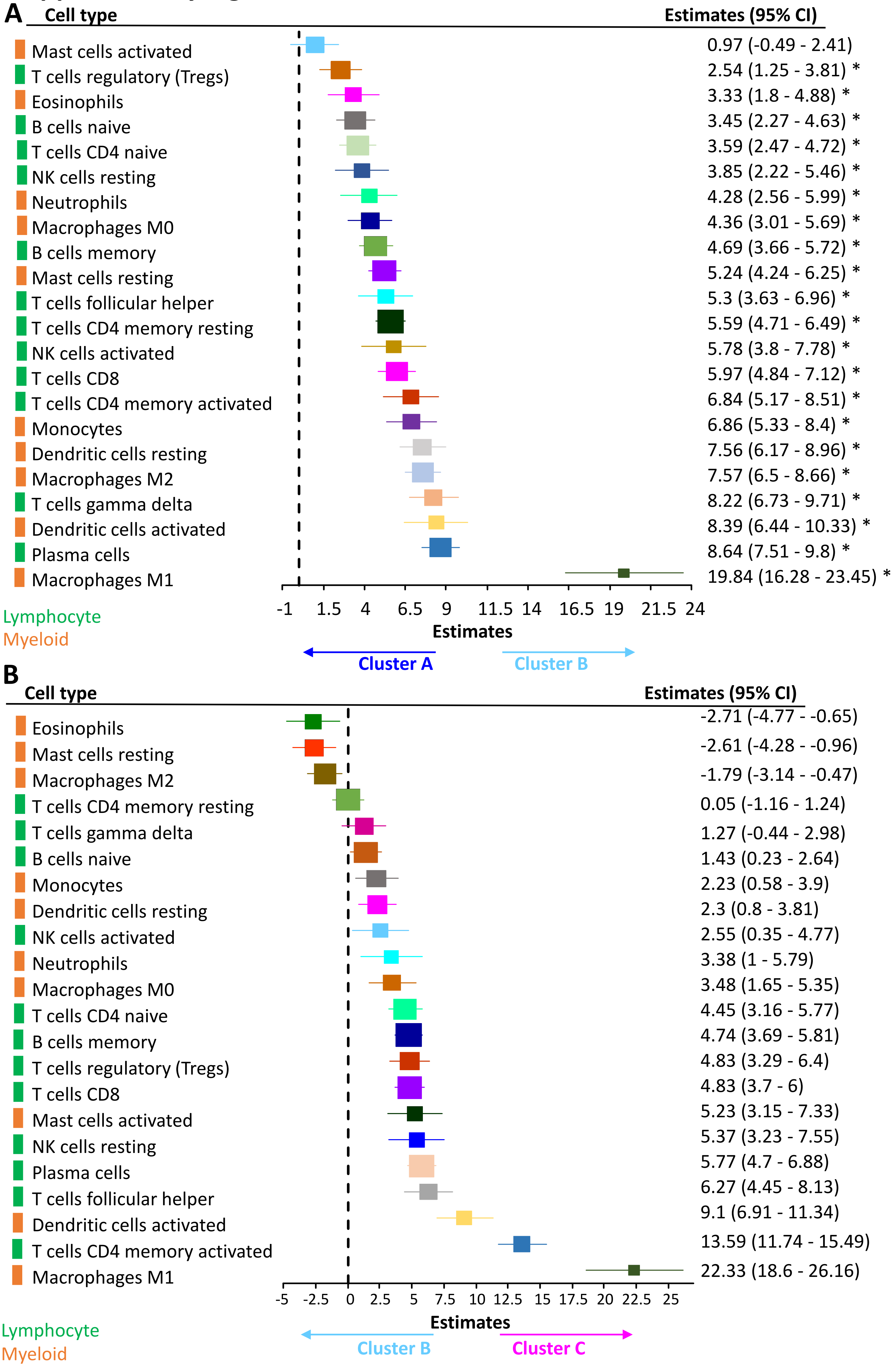

Supplementary Figure 10: Explanatory value of CIBERSORT scores upon comparison of Cluster B versus Cluster A or Cluster C

Estimates of multivariable logistic regression analysis and the 95% Confidence interval (CI) are illustrated by forest plot to assess which immune cell types inferred by CIBERSORT explain the most the poor prognosis cluster (Cluster B). **(A)** Cluster A versus Cluster B, **(B)** Cluster B versus Cluster C. Box size is inversely proportional to the width of the confidence interval. Asterisks denote FDR corrected p-value < 0.05. Immune cell types from the lymphoid or myeloid lineage are identified.

Supplementary Figure 11

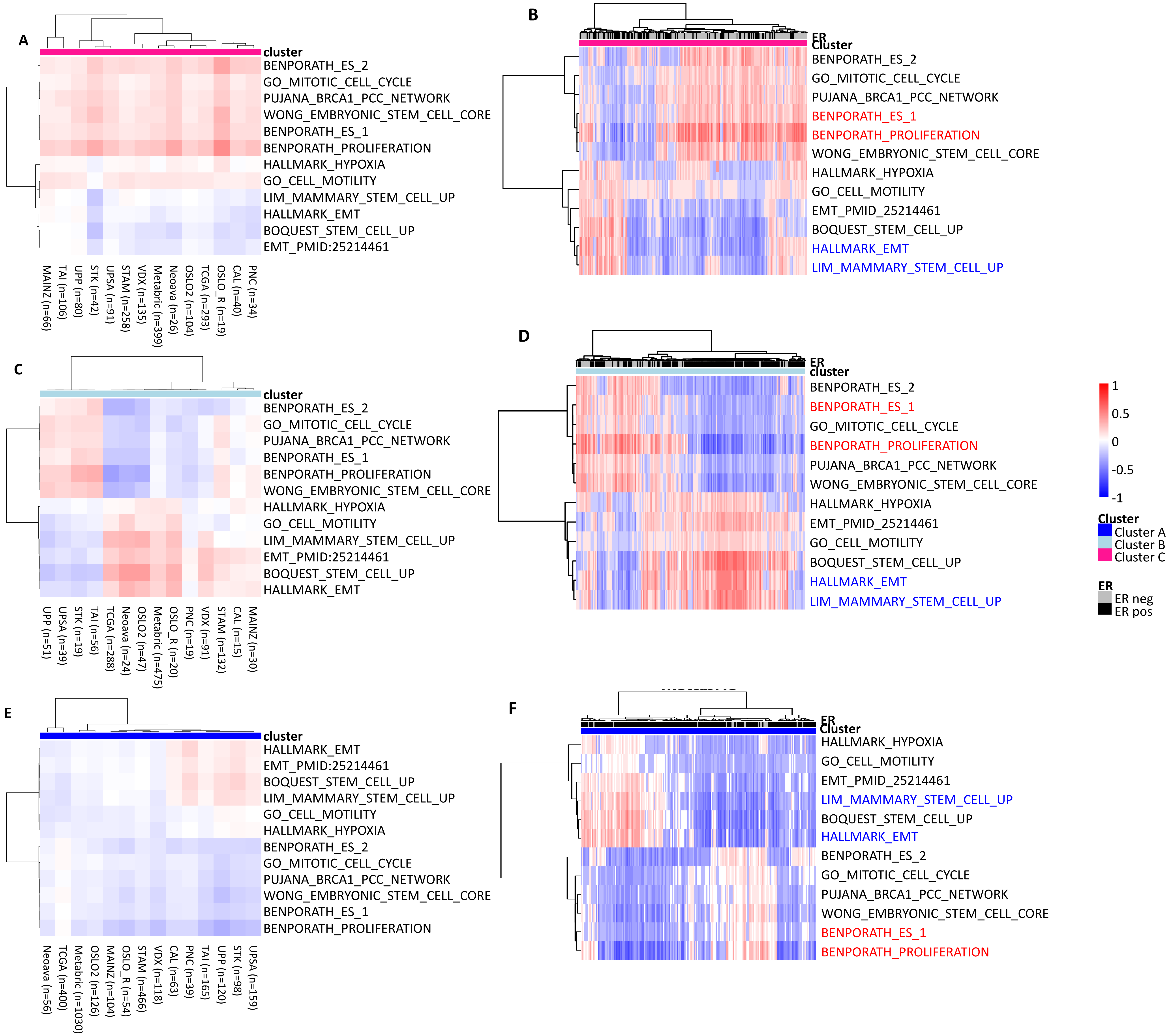

Supplementary Figure 11: Pathway enriched in each immune cluster

Samples were scored using the GSVA Biocoductor package for enrichment in 12 pathways. Average enrichment score is calculated for each immune cluster and cohort, unsupervised clustering using correlation distance and ward.D linkage for each immune cluster was performed. Cluster C (**A**), Cluster B (**C**), Cluster C (**E**). The numbers of samples in each cohort and clusters are denoted.
Unsupervised clustering clustering using correlation distance and ward.D linkage of the pathway enrichment scores for the METABRIC cohort is shown for each cluster. Cluster C (**B**), Cluster B (**D**), Cluster A (**F**).

Supplementary Figure 12

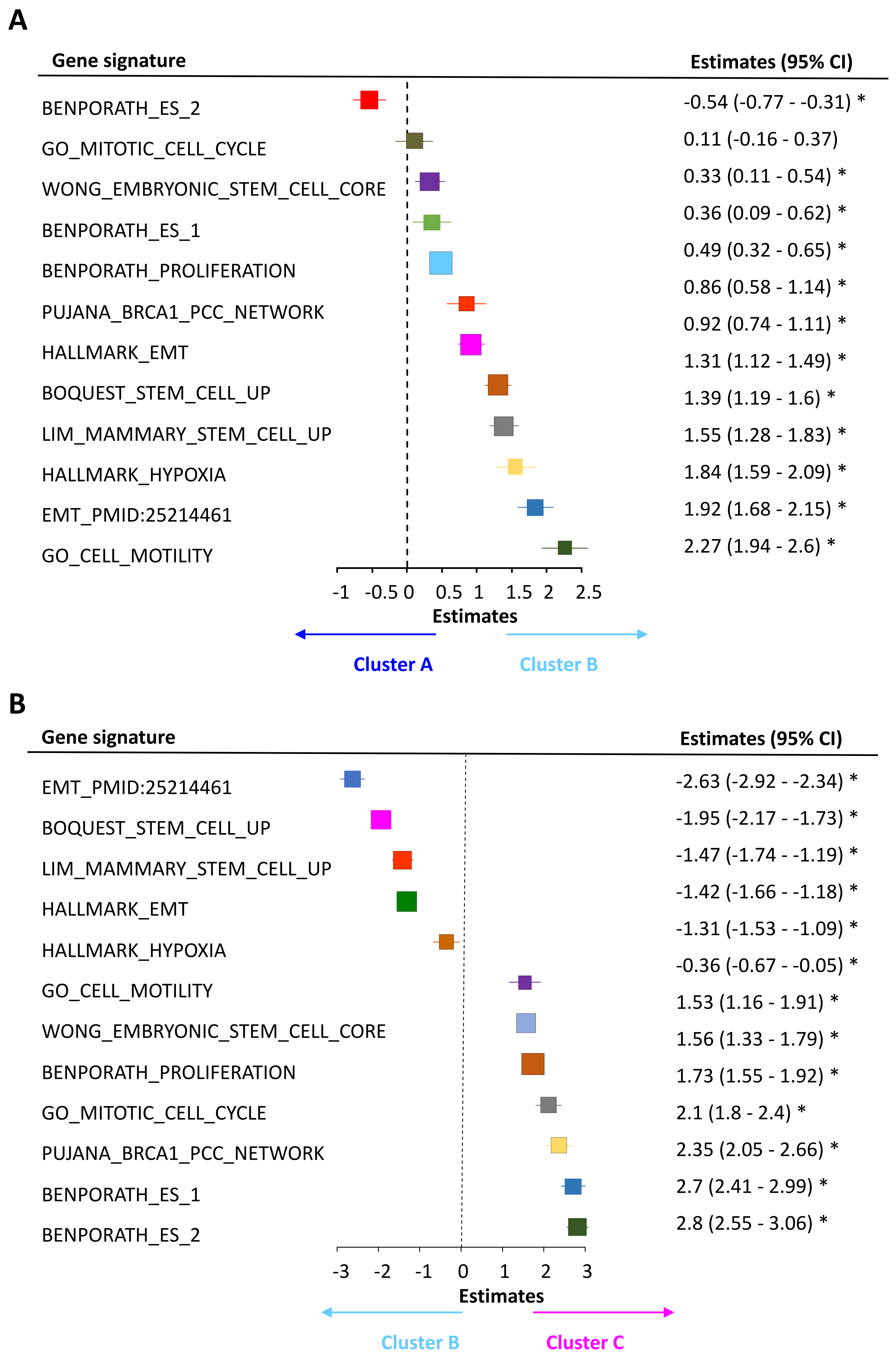

Supplementary Figure 12: Explanatory value of gene-set signature scores upon comparison of Cluster B versus Cluster A or Cluster C

Estimates of univariate logistic regression analysis and the 95% Confidence interval (CI) are illustrated by forest plot to assess which gene-set signature scores calculated using GSVA associate with the poor prognosis cluster. **(A)** Cluster B versus Cluster A, **(B)** Cluster B versus Cluster C. Box size is inversely proportional to the width of the confidence interval. Asterisks denote FDR corrected p-value < 0.05.

Supplementary Figure 13

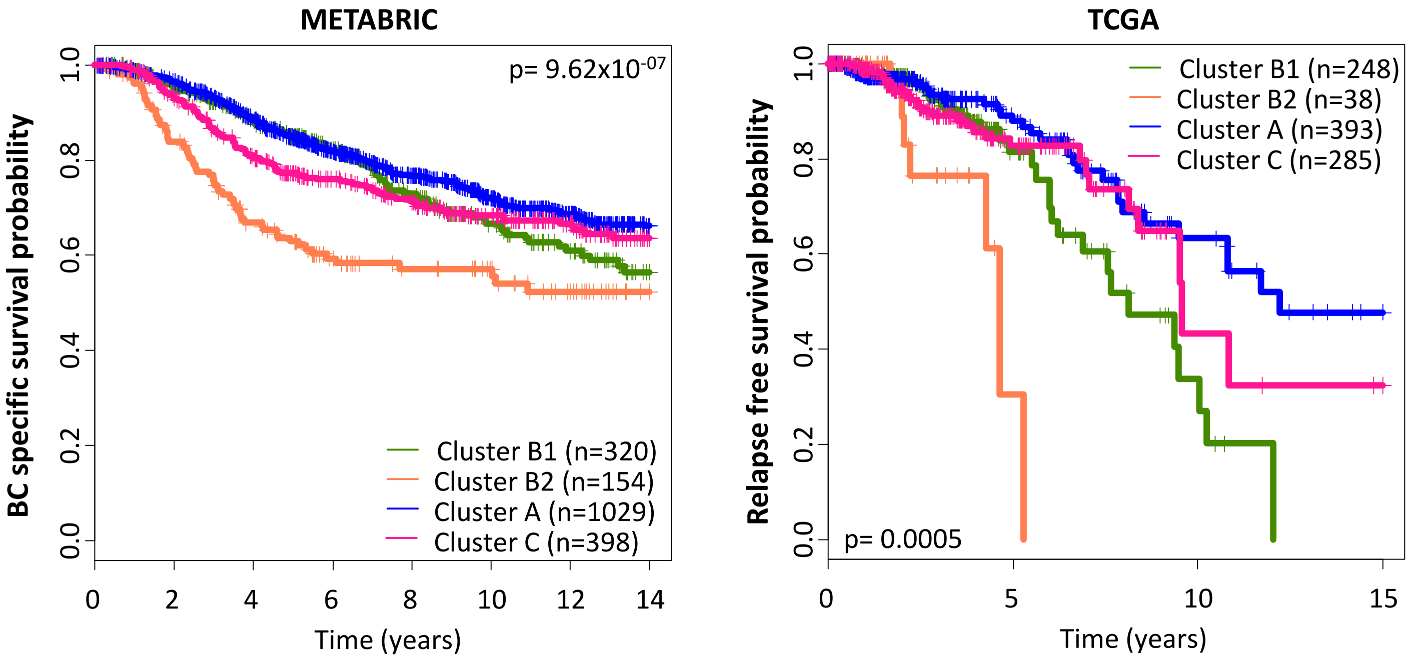

Supplementary Figure 13: Survival analysis across subclusters

Kaplan-Meier survival curves for Cluster A (blue), Cluster C (pink), Cluster B1 (green) and Cluster B2 (orange). In all METABRIC (B) and TCGA (C) samples. The p-values are from log-rank tests. Kaplan-Meier display breast cancer specific survival for the METABRIC and relapse free survival for the TCGA.

Supplementary Figure 14

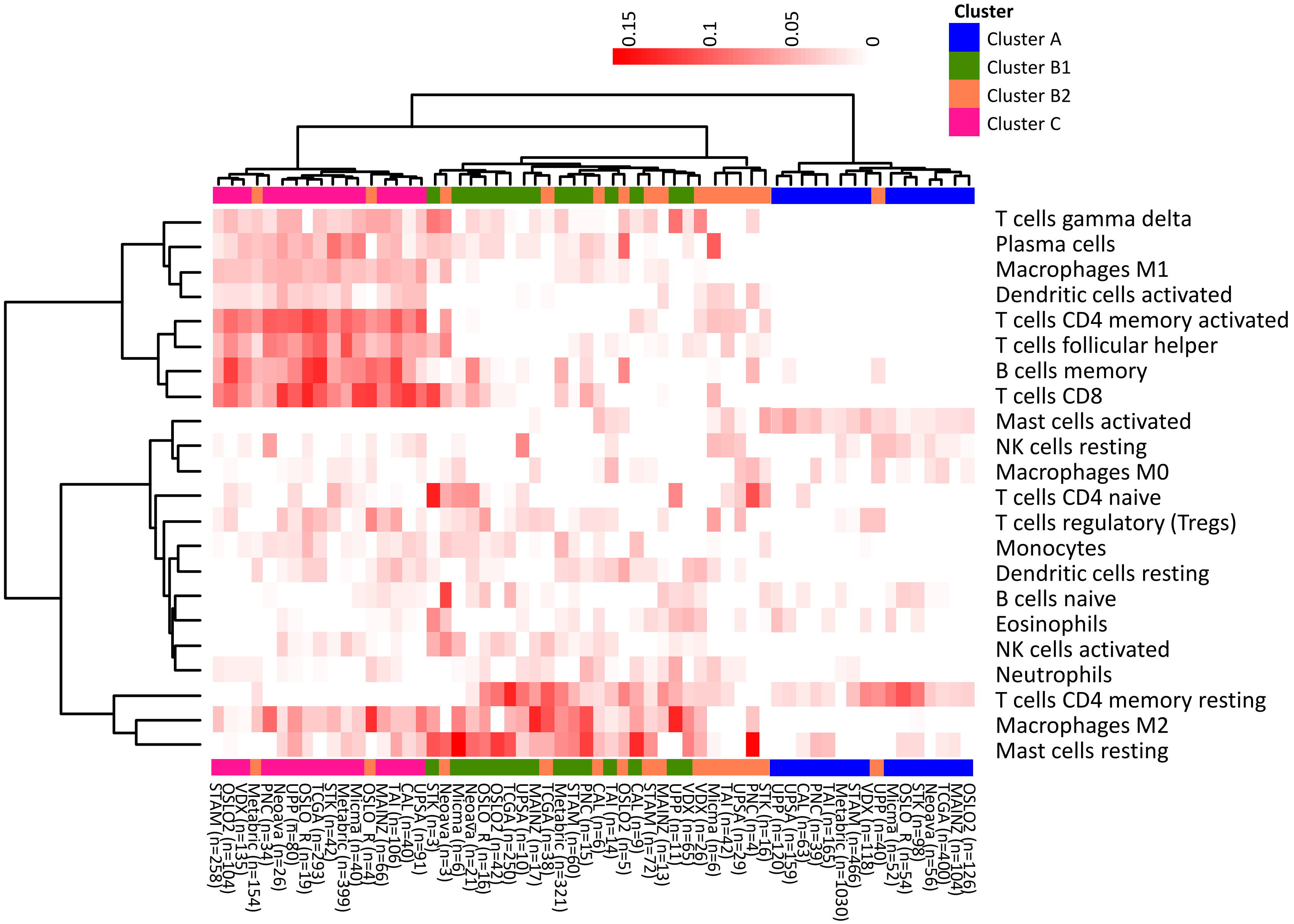

Supplementary Figure 14: Immune infiltration in clusters and subclusters

We used the CIBERSORT algorithm to assess the composition of the immune microenvironment of breast cancer samples. For each cluster and subcluster we calculated the median of the absolute score of the 22 cell types given by the CIBERSORT in each cohort. Cluster-specific-cell-types-medians-scores were used in an unsupervised clustering using maximum distance and the Ward.D2 linkage. The heatmap obtained allows to visualize which cell types are enriched across the immune clusters and subclusters. Immune clusters and subclusters are annotated on the top and bottom of the heatmap.

Supplementary Table 1: Two way table of the cluster assignement using the nanostring data (760 genes) versus the agilent data (509) genes of 79 MicMa samples

		Agilent Cluster assignement (509 genes)		
		Cluster A	Cluster B	Cluster C
**Nanostring Cluster assignement (760 genes)**	Cluster A	31	3	5
	Cluster B	4	5	3
	Cluster C	4	1	23

Fisher exact Test = 1.02x10-8

Supplementary Table 2: Breast cancer cohorts used in this study

Study name	N patients	Platform	Endpoint	Lasso	Accession number/Source	PMID
MAINZ	200	Affymetrix HGU	DMFS	Validation	GEO: GSE11121*	18593943
METABRIC	1904	Illumina HT-12 v3	BCSS	Training	EGAD00010000210	22522925
MicMa	104	Agilent whole genome 4 × 44 K and nCounter	NA	Training	GEO: GSE19536.	21364938 and current study
NeoAva	106	Agilent Sureprint G3 Human GE 8x60k	pCR	Training	AE: E-MTAB-4439.	28487444
Oslo2	277	Agilent SurePrint G3 Human GE 8x60K	NA	Training	GEO: GSE58215	25873999
OSLO2-EMIT0	93	RNA_seq	NA	Training	GEO: GSEXXXX	current study
STAM	856	Affymetrix HGU	RFS	Validation	GEO: GSE6532/GSE9195*	17401012
STK	159	Affymetrix HGU	RFS	Training	GEO: GSE1456*	16280042
TAI	327	Affymetrix HGU	OS	Training	GEO: GSE20685	21501481
TCGA	981	RNA-seq	RFS	Training	TCGA data portal	26451490
UPP	251	Affymetrix HGU	RFS	Training	GEO: GSE3494*	16141321
VDX	344	Affymetrix HGU	DMFS	Training	GEO: GSE2034/GSE5327*	17420468
UPSA	289	Affymetrix HGU	RFS	Validation	GEO: GSE4922*	17079448
PNC	92	Affymetric HGU	OS	Validation	GEO: GSE20711*	21910250
CAL	118	Affymetrix HGU	OS	Validation	AE: E-TABM-158	17157792
MDACC	508	Affymetrix HGU	pCR	NA	GEO: GSE25066	21558518
MDACC TFAC	178	Affymetrix HGU	pCR	NA	GEO: GSE20271	20829329
MDACC IGR	103	Affymetrix HGU	pCR	NA	GEO: GSE22093	21191116
MDACC MAQC	278	Affymetrix HGU	pCR	NA	GEO: GSE20194	20064235
USO	61	Affymetrix HGU	pCR	NA	GEO: GSE23988	21191116
VAN	28	Affymetrix HGU	pCR	NA	GEO: GSE22513	20068102
Osaka	115	Affymetrix HGU	pCR	NA	GEO: GSE32646	22320227

Abbreviations: RFS - relapse free survival; DMFS - distant metastasis-free survival; OS - overall survival; BCSS - breast cancer-specific survival; NA - not available; GEO - Gene Expression Omnibus; AE - array express ; pCR - pathological Complete Response

*Data were accessed from http://compbio.dfci.harvard.edu/pubs/sbtpaper/

Supplementary Table 3: p-values from log-rank tests of the survival analysis using the immune clusters obtained by the clustering or lasso method

		METABRIC			TCGA			STK			UPP			VDX			TAI		
		whole	ER neg	ER pos	whole	ER neg	ER pos	whole	ER neg	ER pos	whole	ER neg	ER pos	whole	ER neg	ER pos	whole	ER neg	ER pos
Clustering	Cluster B (n / event)	555 / 177	154 / 71	401 / 106	416 / 54	30 / 10	298 / 44	27 / 15	7 / 4	20 / 11	94 / 33	17 / 8	94 / 33	100 / 48	11 / 7	89 / 42	68 / 28	35 / 17	33 / 11
	Cluster A & C (n / event)	1346 / 357	289 / 102	1057 / 255	548 / 54	148 /16	284 / 29	132 / 31	13 / 3	119 / 28	140 / 25	26 / 4	140 / 25	244 / 80	124 / 33	120 / 47	259 / 49	45 / 7	214 / 42
	log rank p value	0.0004	0.0130	0.0418	0.0243	0.0045	0.0046	0.0007	0.0819	0.0042	0.0017	0.0075	0.0017	0.0192	0.0129	0.3694	1.32E-05	0.0008	0.0484
Lasso prediction	Cluster B (n / event)	474 / 152	133 / 60	341 / 92	286 / 40	43299	208 / 33	19 /11	6 / 4	13 / 7	49 / 19	9 / 5	49 / 19	91 / 49	13 / 8	78 / 41	56 / 21	28 / 13	28 / 8
	Cluster A & C (n / event)	1427 / 373	310 / 113	1117 / 269	678 / 68	160 / 19	374 / 40	140 / 35	14 / 3	126 / 32	185 / 39	34 / 7	185 / 39	253 / 80	122 / 32	131 / 48	271 / 56	52 / 11	219 / 45
	log rank p value	0.0003	0.0456	0.0127	0.0042	0.0039	0.0004	0.0012	0.0218	0.0215	0.0052	0.0038	0.0052	0.0005	0.0069	0.0315	0.0030	0.0125	0.3651

Supplementary Table 4: Summary statistics of the multivariate cox regression analysis including clinicopathological features as covariates

	TCGA		METABRIC		TAI		VDX		STK		UPP		MAINZ		STAM		UPSA		CAL		PNC	
**Factors**	**HR (95%CI)**	**p-value**	**HR (95%CI)**	**p-value**	**HR (95%CI)**	**p-value**	**HR (95%CI)**	**p-value**	**HR (95%CI)**	**p-value**	**HR (95%CI)**	**p-value**	**HR (95%CI)**	**p-value**	**HR (95%CI)**	**p-value**	**HR (95%CI)**	**p-value**	**HR (95%CI)**	**p-value**	**HR (95%CI)**	**p-value**
**Immune Clusters**																						
Clust B vs Clust A-C	0.33 (0.19 - 0.59)	**0.0001**	0.73 (0.6 - 0.89)	**0.002**	0.64 (0.38 - 1.07)	0.087	0.49 (0.33 - 0.73)	**0.0005**	0.53 (0.24 - 1.18)	0.12	0.5 (0.26 - 0.95)	**0.034**	0.34 (0.16 - 0.73)	**0.006**	0.7 (0.49 - 1)	**0.049**	0.57 (0.33 - 1.01)	**0.05**	0.3 (0.11 - 0.81)	**0.02**	0.56 (0.25 - 1.26)	0.16
**ER status**																						
ER neg vs ER pos	0.6 (0.3 - 1.2)	0.15	0.77 (0.55 - 1.07)	0.11	0.77 (0.37 - 1.63)	0.50	0.96 (0.51 - 1.79)	0.90	3.23 (1.02 - 10.2)	**0.046**	1.37 (0.48 - 3.91)	0.55	2.42 (0.47 - 12.45)	0.29	0.67 (0.27 - 1.65)	0.38	2.37 (1.04 - 5.38)	**0.04**	2.65 (0.51 - 13.9)	0.25	0.42 (0.03 - 6.15)	0.53
**PAM50**																						
Basal-like vs Her2	1.38 (0.55 - 3.44)	0.49	1.21 (0.9 - 1.62)	0.21	2.51 (1.10 - 5.71)	**0.029**	0.81 (0.40 - 1.61)	0.55	1.27 (0.42 - 3.86)	0.67	2.86 (0.94 - 8.71)	0.06	0.62 (0.14 - 2.67)	0.52	0.85 (0.43 - 1.67)	0.64	1.7 (0.72 - 4.03)	0.23	1.52 (0.54 - 4.23)	0.43	2.08 (0.68 - 6.32)	0.20
Basal-like vs LumA	0.69 (0.27 - 1.77)	0.44	0.54 (0.36 - 0.82)	**0.004**	1.05 (0.35 - 3.10)	0.93	0.54 (0.26 - 1.14)	0.11	0.1 (0.02 - 0.43)	**0.002**	1.12 (0.29 - 4.37)	0.87	0.17 (0.02 - 1.15)	0.07	0.56 (0.22 - 1.46)	0.24	0.4 (0.15 - 1.06)	0.07	0.34 (0.05 - 2.3)	0.27	1.48 (0.07 - 30.83)	0.80
Basal-like vs LumB	1.06 (0.43 - 2.64)	0.89	1.13 (0.76 - 1.68)	0.55	2.05 (0.72 - 5.82)	0.18	1.27 (0.62 - 2.58)	0.51	0.57 (0.2 - 1.63)	0.30	1.4 (0.39 - 5.04)	0.61	0.31 (0.05 - 2.08)	0.23	1.41 (0.56 - 3.58)	0.47	0.97 (0.36 - 2.58)	0.95	0.64 (0.11 - 3.79)	0.62	2.76 (0.17 - 45.13)	0.48
Basal-like vsNormal	1.52 (0.55 - 4.21)	0.42	0.94 (0.6 - 1.46)	0.77	3.27 (1.05 - 10.23)	**0.042**	0.22 (0.05 - 0.97)	**0.045**	0.07 (0.01 - 0.69)	**0.022**	1.92 (0.4 - 9.22)	0.42	0.59 (0.08 - 4.24)	0.60	0 (0 - Inf)	0.99	0.62 (0.2 - 1.9)	0.40	0.84 (0.08 - 8.65)	0.88	7.62 (0.86 - 67.31)	0.07
**Lymph node status**																						
Node neg vs Node pos	1.29 (0.69 - 2.42)	0.43	2.13 (1.71 - 2.63)	**< 0.0001**							2.03 (1.13 - 3.66)	**0.018**			1.35 (0.95 - 1.93)	0.10			0.91 (0.49 - 1.68)	0.76		
**Grade**																						
Grade 1 vs Grade 2	0.78 (0.36 - 1.69)	0.53	1.38 (0.85 - 2.24)	0.19					1.22 (0.38 - 3.92)	0.74	1.94 (0.82 - 4.64)	0.13	3.05 (0.68 - 13.68)	0.15								
Grade 1 vs Grade 3	0.7 (0.25 - 1.94)	0.50	1.58 (0.97 - 2.57)	0.07					0.76 (0.22 - 2.67)	0.67	2.91 (1.03 - 8.17)	**0.043**	5.24 (1 - 27.48)	0.05								
**Stage**																						
Stage 1 vs Stage 2	1.8 (0.62 - 5.26)	0.28	0.77 (0.61 - 0.97)	**0.029**																		
Stage 1 vs Stage 3	2.02 (0.5 - 8.12)	0.32	1.39 (0.97 - 1.98)	0.070																		
Stage 1 vs Stage 4	5.31 (1.2 - 23.51)	**0.028**	3.36 (1.54 - 7.33)	**0.002**																		
**Age**																						
Continuous variable	1.04 (1.02 - 1.06)	**< 0.0001**	1.01 (1 - 1.02)	0.05	0.99 (0.97 - 1.01)	0.45	0.99 (0.97 - 1.00)	0.138826	1 (0.97 - 1.03)	0.97	1.01 (0.99 - 1.03)	0.32	1 (0.97 - 1.04)	0.78	0.99 (0.98 - 1.01)	0.26	1 (0.98 - 1.02)	0.99	1.01 (0.99 - 1.03)	0.55	1.39 (0.64 - 3)	0.41
**Tumor size**																						
Continuous variable			1.01 (1 - 1.01)	**0.0002**			1.01 (0.74 - 1.38)	0.940281			1.44 (1.13 - 1.82)	**0.003**	1.29 (0.94 - 1.79)	0.12	1.01 (0.9 - 1.13)	0.86	1.01 (1 - 1.02)	**0.04**	0.93 (0.71 - 1.2)	0.56	1.09 (0.9 - 1.33)	0.38

Abbreviation: HR, hazard ratio; 95%CI, 95% confidence interval
p values are from Cox proportional hazards model

Supplementary Table 5 : Akaike information criterion (AIC) index from cox multivariable cox regression analysis using all relevant variable available for each cohort with or without immune clusters as a variable

	METABRIC	TCGA	TAI	VDX	STK	UPP	MAINZ	STAM	UPSA	CAL	PNC
**AIC index all variables**	7246.4	853.2	892.9	1260.2	380.5	558.0	405.4	2228.5	918.3	448.4	262.0
**AIC index without immune clusters**	7252.4	865.9	893.7	1269.9	380.8	560.2	410.2	2230.2	919.7	451.7	261.9

Supplementary Table 6 : Summary statistics of the multivariate cox regression analysis, comparing the ROR score to immune clusters

Multivariable cox regression analysis including ROR scores and immune clusters

	TCGA		METABRIC		TAI		VDX		STK		UPP	
	Relapse free survival		Breast cancer specific survival		Overall survival		Distant metastasis free survival		Relapse free survival		Relapse free survival	
Factors	HR (95%CI)	p-value	HR (95%CI)	p-value	HR (95%CI)	p-value	HR (95%CI)	p-value	HR (95%CI)	p-value	HR (95%CI)	p-value
Immune Clusters												
	0.66 (0.54 - 0.79)	**< 0.0001**	0.35 (0.21 - 0.58)	**0.0001**	0.69 (0.41 - 1.16)	0.1639	0.42 (0.29 - 0.61)	**< 0.0001**	0.46 (0.23 - 0.92)	**0.0284**	0.57 (0.33 - 0.99)	**0.0461**
ROR score												
	10.94 (7.15 - 16.74)	**< 0.0001**	4.34 (1.7 - 11.06)	**0.0021**	4.25 (1.6 - 11.32)	**0.0037**	4.45 (2.04 - 9.71)	**0.0002**	9.42 (2.57 - 34.46)	**0.0007**	7.79 (2.52 - 24.12)	**0.0004**

	MAINZ		STAM		UPSA		CAL		PNC	
	Distant metastasis free survival		Relapse free survival		Relapse free survival		Relapse free survival		Relapse free survival	
Factors	HR (95%CI)	p-value	HR (95%CI)	p-value	HR (95%CI)	p-value	HR (95%CI)	p-value	HR (95%CI)	p-value
Immune Clusters										
	0.49 (0.25 - 0.99)	**0.0471**	0.71 (0.51 - 1)	**0.0478**	0.58 (0.34 - 0.99)	**0.0464**	0.3 (0.15 - 0.6)	**0.0006**	0.49 (0.24 - 1.01)	0.0522
ROR score										
	7.54 (2.01 - 28.25)	**0.0027**	8.92 (4.97 - 16.03)	**< 0.0001**	5 (2.16 - 11.59)	**0.0002**	2.07 (0.68 - 6.34)	0.2023	2.52 (0.59 - 10.79)	0.2136

NRI and IDI indexes comparing ROR scores and immune clusters

	TCGA		METABRIC		TAI		VDX		STK		UPP	
	Relapse free survival		Breast cancer specific survival		Overall survival		Distant metastasis free survival		Relapse free survival		Relapse free survival	
Factors	Estimate	p-value	Estimate	p-value	Estimate	p-value	Estimate	p-value	Estimate	p-value	Estimate	p-value
IDI	0.07 (-0.27 - 0.43)	0.2458	0.02 (0.01 - 0.04)	**0.0080**	0.01 (0 - 0.05)	0.0619	0.02 (0 - 0.09)	**0.04795205**	0.04 (0 - 0.13)	**0.0380**	0.02 (0 - 0.09)	**0.0420**
NRI	0.58 (-0.91 - 1.81)	0.2018	0.14 (0.07 - 0.23)	**0.0120**	0.06 (-0.11 - 0.23)	0.3936	0.18 (-0.2 - 0.34)	0.27972028	0.18 (-0.27 - 0.33)	0.2637	0.17 (-0.06 - 0.32)	0.0979

	MAINZ		STAM		UPSA		CAL		PNC	
	Distant metastasis free survival		Relapse free survival		Relapse free survival		Relapse free survival		Relapse free survival	
Factors	Estimate	p-value	Estimate	p-value	Estimate	p-value	Estimate	p-value	Estimate	p-value
IDI	0.02 (0 - 0.09)	**0.0480**	0.01 (0 - 0.03)	**0.0460**	0.03 (0 - 0.09)	0.0639	0.06 (0.02 - 0.11)	**0.0080**	0.08 (-0.03 - 0.26)	0.1019
NRI	0.18 (-0.2 - 0.34)	0.2797	0.1 (-0.06 - 0.18)	0.0859	0.19 (-0.12 - 0.28)	0.1239	0.18 (0.07 - 0.33)	**0.0180**	0.32 (-0.23 - 0.79)	0.1578

Abbreviation: HR, hazard ratio; 95%CI, 95% confidence interval, NRI, net reclassification improvement, IDI, integrated discrimination improvemen
p values are from Cox proportional hazards model

Supplementary Table 7: Chi-square p-values assessing the distribution of the samples across immune cluster and response to neoadjuvant chemotherapy in the whole cohort, ER positive and ER negative cases separately

cohort	All samples	ER positif	ER negatif
**MDACC**	**< 0.0001**	0.1500	**0.0324**
**MDACC_IGR**	**0.0222**	0.3831	**0.0009**
**MDACC_TFAC**	**0.0124**	0.2865	0.1479
**MDACC_MAQC**	**0.0012**	0.7880	0.9424
**Osaka**	**0.0001**	0.1104	**0.0438**
**USO**	**0.0342**	0.4809	0.0767
**Neoava**	**0.0006**	0.2154	0.1814
**VAN**	**0.0215**	0.1088	NA
**All cohorts**	**< 0.0001**	**< 0.0001**	**0.0003**

Supplementary Table 8: Summary statistics of the multivariate cox regression analysis, comparing the Lympho score to immune clusters

	TCGA		METABRIC		TAI		VDX		STK		UPP	
	Relapse free survival		Breast cancer specific survival		Overall survival		Distant metastasis free survival		Relapse free survival		Relapse free survival	
Factors	HR (95%CI)	p-value	HR (95%CI)	p-value	HR (95%CI)	p-value	HR (95%CI)	p-value	HR (95%CI)	p-value	HR (95%CI)	p-value
Immune Clusters												
	0.71 (0.58 - 0.86)	**0.0005**	0.57 (0.38 - 0.86)	**0.0073**	0.56 (0.34 - 0.92)	**0.0213**	0.55 (0.39 - 0.79)	**0.0010**	0.36 (0.18 - 0.72)	**0.0039**	0.46 (0.25 - 0.85)	**0.0129**
Lympho score												
	1 (0.84 - 1.19)	0.9779	1.03 (0.84 - 1.26)	0.7892	0.7 (0.52 - 0.95)	**0.0230**	0.63 (0.47 - 0.85)	**0.0023**	0.7 (0.33 - 1.5)	0.3628	1 (0.66 - 1.52)	0.9872

	MAINZ		STAM		UPSA		CAL		PNC	
	Distant metastasis free survival		Relapse free survival		Relapse free survival		Relapse free survival		Relapse free survival	
Factors	HR (95%CI)	p-value	HR (95%CI)	p-value	HR (95%CI)	p-value	HR (95%CI)	p-value	HR (95%CI)	p-value
Immune Clusters										
	0.44 (0.22 - 0.89)	**0.0221**	0.7 (0.5 - 0.98)	**0.0383**	0.45 (0.26 - 0.79)	**0.0048**	0.32 (0.16 - 0.63)	**0.0009**	0.54 (0.26 - 1.1)	0.0914
Lympho score										
	0.74 (0.47 - 1.17)	0.1938	0.81 (0.65 - 1.01)	0.0556	1.05 (0.78 - 1.43)	0.7313	1.04 (0.73 - 1.46)	0.8434	0.83 (0.53 - 1.32)	0.4357

Abbreviation: HR, hazard ratio; 95%CI, 95% confidence interval
p values are from Cox proportional hazards model

Supplementary Table 9: Summary statistics of the uni - and multivariate cox regression analysis, comparing the EMT score form the HALLMARK-EMT gene set to immune clusters

	TCGA		METABRIC		TAI		VDX		STK		UPP	
	RFS		BCSS		Overall survival		DMFS		RFS		RFS	
Factors	HR (95%CI)	p-value	HR (95%CI)	p-value	HR (95%CI)	p-value	HR (95%CI)	p-value	HR (95%CI)	p-value	HR (95%CI)	p-value
Immune Clusters												
	0.52 (0.35 - 0.79)	**0.002**	0.68 (0.56 - 0.83)	**< 0.0001**	0.5 (0.3 - 0.82)	0.006	0.56 (0.39 - 0.81)	**0.002**	0.39 (0.2 - 0.78)	0.008	0.52 (0.29 - 0.91)	0.022
HALLMARK_EMT												
	0.66 (0.38 - 1.13)	0.126	0.82 (0.63 - 1.06)	0.128	1.62 (0.87 - 2.98)	0.126	1.3 (0.77 - 2.21)	0.326	0.45 (0.18 - 1.1)	0.081	0.59 (0.27 - 1.26)	0.175

	MAINZ		STAM		UPSA		CAL		PNC	
	DMFS		RFS		RFS		RFS		RFS	
Factors	HR (95%CI)	p-value	HR (95%CI)	p-value	HR (95%CI)	p-value	HR (95%CI)	p-value	HR (95%CI)	p-value
Immune Clusters										
	0.38 (0.19 - 0.76)	0.006	0.7 (0.5 - 0.97)	0.033	0.49 (0.29 - 0.84)	0.010	0.33 (0.17 - 0.63)	**0.001**	0.51 (0.25 - 1.05)	0.068
HALLMARK_EMT										
	0.25 (0.1 - 0.65)	**0.004**	0.99 (0.68 - 1.44)	0.951	0.7 (0.39 - 1.26)	0.234	0.82 (0.4 - 1.71)	0.601	1.74 (0.71 - 4.25)	0.228

	TCGA		METABRIC		TAI		VDX		STK		UPP	
	RFS		BCSS		Overall survival		DMFS		RFS		RFS	
Factors	HR (95%CI)	p-value	HR (95%CI)	p-value	HR (95%CI)	p-value	HR (95%CI)	p-value	HR (95%CI)	p-value	HR (95%CI)	p-value
HALLMARK_EMT												
	0.93 (0.73 - 1.19)	0.568	0.78 (0.46 - 1.31)	0.345	1.46 (0.79 - 2.68)	0.229	1.55 (0.93 - 2.58)	0.096	0.38 (0.16 - 0.91)	0.030	0.48 (0.23 - 1.02)	0.055

	MAINZ		STAM		UPSA		CAL		PNC	
	DMFS		RFS		RFS		RFS		RFS	
Factors	HR (95%CI)	p-value	HR (95%CI)	p-value	HR (95%CI)	p-value	HR (95%CI)	p-value	HR (95%CI)	p-value
HALLMARK_EMT										
	0.28 (0.11 - 0.72)	0.008	1.01 (0.7 - 1.47)	0.948	0.63 (0.35 - 1.13)	0.120	0.81 (0.39 - 1.69)	0.571	1.68 (0.68 - 4.11)	0.258
