# Supplementary material for: An independent poor-prognosis subtype of breast cancer defined by a distinct tumor immune microenvironment
Source: Nat Commun. 2019 Dec 3;10:5499. doi: 10.1038/s41467-019-13329-5 (PMC6890706; doi:10.1038/s41467-019-13329-5)
Supplement: Supplementary file 3 — Description of Additional Supplementary Files [file 41467_2019_13329_MOESM3_ESM.pdf]

**Description of Additional Supplementary Files**

File Name: Supplementary Data 1
Description: Output genes of the lasso analysis

File Name: Supplementary Data 2
Description: Results of the differential expression analysis between the immune clusters

File Name: Supplementary Data 3
Description: Pathways investigated in this study using GSVA and genes associated

File Name: Supplementary Data 4
Description: Output genes of the Nanodissect analysis

File Name: Supplementary Data 5
Description: Normalized nCounter counts for the FFPE MicMa samples

:
