# Supplementary material for: An independent poor-prognosis subtype of breast cancer defined by a distinct tumor immune microenvironment
Source: Nat Commun. 2019 Dec 3;10:5499. doi: 10.1038/s41467-019-13329-5 (PMC6890706; doi:10.1038/s41467-019-13329-5)
Supplement: Supplementary file 9 — Reporting Summary [file 41467_2019_13329_MOESM9_ESM.pdf]

nature research

Corresponding author(s): Vessela Kristensen

Last updated by author(s): Sep 12, 2019

Reporting Summary

Nature Research wishes to improve the reproducibility of the work that we publish. This form provides structure for consistency and transparency in reporting. For further information on Nature Research policies, see Authors & Referees and the Editorial Policy Checklist.

Statistics

For all statistical analyses, confirm that the following items are present in the figure legend, table legend, main text, or Methods section.

n/a	Confirmed	
☐	☒	The exact sample size (*n*) for each experimental group/condition, given as a discrete number and unit of measurement
☐	☒	A statement on whether measurements were taken from distinct samples or whether the same sample was measured repeatedly
☐	☒	The statistical test(s) used AND whether they are one- or two-sided<br>*Only common tests should be described solely by name; describe more complex techniques in the Methods section.*
☐	☒	A description of all covariates tested
☐	☒	A description of any assumptions or corrections, such as tests of normality and adjustment for multiple comparisons
☐	☒	A full description of the statistical parameters including central tendency (e.g. means) or other basic estimates (e.g. regression coefficient) AND variation (e.g. standard deviation) or associated estimates of uncertainty (e.g. confidence intervals)
☐	☒	For null hypothesis testing, the test statistic (e.g. *F*, *t*, *r*) with confidence intervals, effect sizes, degrees of freedom and *P* value noted<br>*Give P values as exact values whenever suitable.*
☒	☐	For Bayesian analysis, information on the choice of priors and Markov chain Monte Carlo settings
☐	☒	For hierarchical and complex designs, identification of the appropriate level for tests and full reporting of outcomes
☒	☐	Estimates of effect sizes (e.g. Cohen's *d*, Pearson's *r*), indicating how they were calculated

Our web collection on statistics for biologists contains articles on many of the points above.

Software and code

Policy information about availability of computer code

Data collection	Data used in the study are clearly defined as well as if additional normalizations have been performed before downstream analysis
Data analysis	All codes are available, either at the Source Data file and /or on a GitHub web page as described in the manuscript. This will allow readers to test the methods in their own data sets or in a specific question setting. A web interface is also maintained by the authors

For manuscripts utilizing custom algorithms or software that are central to the research but not yet described in published literature, software must be made available to editors/reviewers. We strongly encourage code deposition in a community repository (e.g. GitHub). See the Nature Research guidelines for submitting code & software for further information.

Data

Policy information about availability of data

All manuscripts must include a data availability statement. This statement should provide the following information, where applicable:

- Accession codes, unique identifiers, or web links for publicly available datasets
- A list of figures that have associated raw data
- A description of any restrictions on data availability

All data used in this study are publicly available or can be downloaded through the European Genome-phenome Archive (EGA) - EMBL-EBI portal. We mainly used gene expression data sets from breast cancers in this study summarized in Supplementary Table 1. Data where downloaded with the study specific normalization process. For initial clustering of the correlation matrix no other normalization was performed. Further on, to produce the single sample predictor and further downstream analysis (CIBERSORT, GSVA, differential expression) the datasets are mean centered.
To reproduce all figures and supplementary figures, publish in this study, we provide all codes and data in a source data file as well as detailed explanations.
The newly generated raw RNA-seq gene expression data for the breast cancer cohort OSLO2-EMIT0 is available at EGA with accession number EGAS00001003631 while the FPKM are available at GEO, GSE135298.
Newly generated, normalized log 2 transformed nCounter counts for the MicMa cohorts can be found in Supplementary Table 12.

Field-specific reporting

Please select the one below that is the best fit for your research. If you are not sure, read the appropriate sections before making your selection.

☒ Life sciences ☐ Behavioural & social sciences ☐ Ecological, evolutionary & environmental sciences

For a reference copy of the document with all sections, see nature.com/documents/nr-reporting-summary-flat.pdf

Life sciences study design

All studies must disclose on these points even when the disclosure is negative.

Sample size	In the present study, we use several breast cancer cohorts and repeatedly found independently of the sample size, the same results.
Data exclusions	No data exclusion
Replication	In the present study, we use several breast cancer cohorts and repeatedly found independently of the sample size, the same results.<br>While, using a machine learning approach to obtain a single sample predictor to obtain the immune cluster an appropriate validation set was used.
Randomization	All breast cancers analyzed in the study are primary breast cancers, no randomization was used here.
Blinding	N/A

Reporting for specific materials, systems and methods

We require information from authors about some types of materials, experimental systems and methods used in many studies. Here, indicate whether each material, system or method listed is relevant to your study. If you are not sure if a list item applies to your research, read the appropriate section before selecting a response.

Materials & experimental systems

n/a	Involved in the study	
☒	☐	Antibodies
☒	☐	Eukaryotic cell lines
☒	☐	Palaeontology
☒	☐	Animals and other organisms
☐	☒	Human research participants
☒	☐	Clinical data

Methods

n/a	Involved in the study	
☒	☐	ChIP-seq
☒	☐	Flow cytometry
☒	☐	MRI-based neuroimaging

Human research participants

Policy information about studies involving human research participants

Population characteristics	For each breast cancer cohorts analyzed we collected the relevant covariates known to associate with prognosis and included these covariates in a multivariate regression analysis.<br>These co/variables were (age, tumor size, node involvement, grade, stage, ER status, PAM50 subtyping)
Recruitment	In the case of our newly published breast cancer cohort: the OSLO2-EMIT0 is a consecutive study collecting material from breast cancer patients with primary operable disease in several hospitals in south eastern Norway. Inclusion of patients started in 2006 and is still on-going.<br>For other publicly available data, please see the relevant publication.
Ethics oversight	The study was approved by the Norwegian Regional Committee for Medical Research Ethics (approval number 1.2006.1607, amendment 1.2007.1125), and patients have given written consent for the use of material for research purposes

Note that full information on the approval of the study protocol must also be provided in the manuscript.
